# Extract from the Marine Seaweed *Padina pavonica* Protects Mitochondrial Biomembranes from Damage by Amyloidogenic Peptides

**DOI:** 10.3390/molecules26051444

**Published:** 2021-03-07

**Authors:** Mario Caruana, Angelique Camilleri, Maria Ylenia Farrugia, Stephanie Ghio, Michaela Jakubíčková, Ruben J. Cauchi, Neville Vassallo

**Affiliations:** 1 Department of Physiology and Biochemistry, Faculty of Medicine and Surgery, University of Malta, 2023 Msida, Malta; mario.caruana@um.edu.mt (M.C.); angleique.camilleri.03@um.edu.mt (A.C.); maria-ylenia.farrugia.12@um.edu.mt (M.Y.F.); stephanie.ghio.00@um.edu.mt (S.G.); misajakubickova@centrum.cz (M.J.); ruben.cauchi@um.edu.mt (R.J.C.); 2 Centre for Molecular Medicine and Biobanking, Biomedical Sciences Building, University of Malta, 2023 Msida, Malta; 3 Department of Experimental Biology, Faculty of Science, Masaryk University, 60300 Brno, Czech Republic

**Keywords:** *Padina pavonica* seaweed extract, Alzheimer’s disease, Parkinson’s disease, mitochondria, amyloidogenic proteins, membrane permeabilization

## Abstract

The identification of compounds which protect the double-membrane of mitochondrial organelles from disruption by toxic confomers of amyloid proteins may offer a therapeutic strategy to combat human neurodegenerative diseases. Here, we exploited an extract from the marine brown seaweed *Padina pavonica* (PPE) as a vital source of natural bioactive compounds to protect mitochondrial membranes against insult by oligomeric aggregates of the amyloidogenic proteins amyloid-β (Aβ), α-synuclein (α-syn) and tau, which are currently considered to be major targets for drug discovery in Alzheimer’s disease (AD) and Parkinson’s disease (PD). We show that PPE manifested a significant inhibitory effect against swelling of isolated mitochondria exposed to the amyloid oligomers, and attenuated the release of cytochrome *c* from the mitochondria. Using cardiolipin-enriched synthetic lipid membranes, we also show that dye leakage from fluorophore-loaded vesicles and formation of channel-like pores in planar bilayer membranes are largely prevented by incubating the oligomeric aggregates with PPE. Lastly, we demonstrate that PPE curtails the ability of Aβ42 and α-syn monomers to self-assemble into larger β-aggregate structures, as well as potently disrupts their respective amyloid fibrils. In conclusion, the mito-protective and anti-aggregator biological activities of *Padina pavonica* extract may be of therapeutic value in neurodegenerative proteinopathies, such as AD and PD.

## 1. Introduction

Neurodegenerative proteinopathies represent a range of devastating medical disorders collectively defined by the accumulation and deposition of protein aggregates in the brain and spinal cord. Examples of peptides or proteins forming fibrillar deposits in the central nervous system include amyloid-β (Aβ) in Alzheimer’s disease (AD), α-synuclein (α-syn) in Parkinson’s disease (PD), tau in frontotemporal lobar degeneration (FTD), and TDP-43 in amyotrophic lateral sclerosis (ALS) [1,2]. Intriguingly, although there is no overt similarity among these amyloidogenic proteins in structure or function, they all feature regions which are prone to significant structural disorder and which predispose the native protein to aberrant folding and assembly into highly toxic aggregates [3]. Increasing evidence suggests that the latter are represented by the soluble clusters that form initially during aggregation, known as oligomers [4]. It is believed that the combination of a small size and a high degree of hydrophobic surface exposure make oligomeric aggregate species highly promiscuous for deleterious interaction with other molecular targets in the neuron or synapse [5,6,7]. These targets include membrane proteins, particularly calcium channels, as well as the phospholipid component of membranes [8,9]. Indeed, direct damage to biomembranes by amyloidogenic proteins is the mechanism most commonly observed when neurodegenerative proteinopathies are addressed in vitro [10,11,12,13,14].

In neurons, mitochondria are particularly abundant to enable synaptic communication by providing large amounts of energy (in the form of adenosine triphosphate, ATP) and calcium buffering [15]. Maintenance of the integrity of the mitochondrial envelope, consisting of outer and inner mitochondrial membranes, is critical for ensuring that mitochondria are able to meet their functional demands. Multiple lines of evidence have especially highlighted mitochondria, and the mitochondrial double-membrane, as potentially important targets of amyloid toxicity [16,17,18,19,20]. Mitochondrial dysfunction leads to disruptive electron transport and generation of reactive oxygen species, culminating in the release of proapoptotic factors such a cytochrome *c* (cyto *c*) and activation of caspases, which designates a point of no return in the apoptotic program. Synaptic impairment and neuronal loss ensue, thereby fueling the neurodegenerative process [21,22]. Therefore, the identification of natural or synthetic compounds which protect mitochondrial membranes from amyloid oligomer-induced disruption may offer a therapeutic strategy to combat human neurodegenerative diseases.

*Padina pavonica* is a marine brown seaweed, also known as Peacock’s tail that inhabits warm temperate to tropical locations, including the Indian, Pacific and Atlantic oceans and also the Mediterranean sea [23]. In recent years, the exploitation of marine algae as a vital source of bioactive compounds has attracted a lot of interest in the pharmaceutical and functional food industries [24,25]. Brown algae are a rich source of compounds and secondary metabolites such as peptides, polyphenols, phytosterols, carotenoids, fatty acids, and polysaccharides [26]. The chemical composition of *Padina pavonica* extract (PPE) as prepared in this study by the Institute of Cellular Pharmacology (Mosta Technopark, Malta) was recently explored by spectrophotometry (for total phenolic, flavonoid and tannin content) and gas chromatography-mass spectrometry (GC-MS) analysis (for lipids and sterol profiles). PPE was found to be especially rich in phenolic compounds, one of the most important classes of natural bioactive compounds: flavonoids and tannins comprised 54.8 mg and 54.4 mg per g of extract, respectively. Hydrocarbons were another major component, with 68.83% corresponding to fatty acids, while sterols represented 8.37% of the extract, including fucosterol and cholesterol [27,28]. It has been previously shown that seaweed-derived compounds, such as phytosterols, are able to cross the blood-brain barrier and accumulate in the central nervous system (CNS), and may therefore exhibit neuromodulatory and neuroprotective properties [29]. For instance, the acetone extract of *Padina gymnospora*, a closely related brown macroalga, has been reported to possess anti-amyloidogenic properties, with in vitro inhibition of aggregation of the Alzheimer’s Aβ fragment 25–35, as well as disaggregation of mature fibrils [30]. A follow-up study revealed anti-oxidant and anti-apoptotic activities in Neuro2a cells, while in an in vivo transgenic *Caenorhabditis elegans* worm model of AD, *P. gymnaspora* extract and its active constituent α-bisabolol antagonized the development of AD-related pathways and macromolecular damage [31]. Lately, we demonstrated in vivo neuroprotective properties for acetone extract from *Padina pavonica*. In *Drosophila melanogaster* flies with brain-specific expression of wild-type Aβ 1–42 (Aβ42; fly model of AD) and the human α-syn A53T mutant (fly model of PD), supplementation of food with PPE dramatically ameliorated lifespan and behavioral signs in the transgenic flies [32]. We also obtained evidence that oligomeric aggregates exposed to PPE were less effective at compromising lipid membranes [32]. In an effort to further our understanding of the mechanisms of action of PPE against amyloid neurodegeneration at the molecular level, in the present study we specifically addressed the potential of PPE to protect mitochondrial membranes. With this aim, we made use of well-established in vitro model systems, comprising synthetic membranes that closely mimic the mitochondrial lipid environment, and isolated mitochondrial organelles [16,17,33]. Overall, we show for the first time that PPE maintains the integrity of mitochondrial lipid membranes in the face of insult from oligomers of different amyloid proteins (Aβ42, α-syn and tau) and hence protects mitochondria from dysfunction.

## 2. Results

### 2.1. PPE Inhibits Amyloid Aggregate-Induced Damage to Isolated Mitochondria

**Figure 1:**
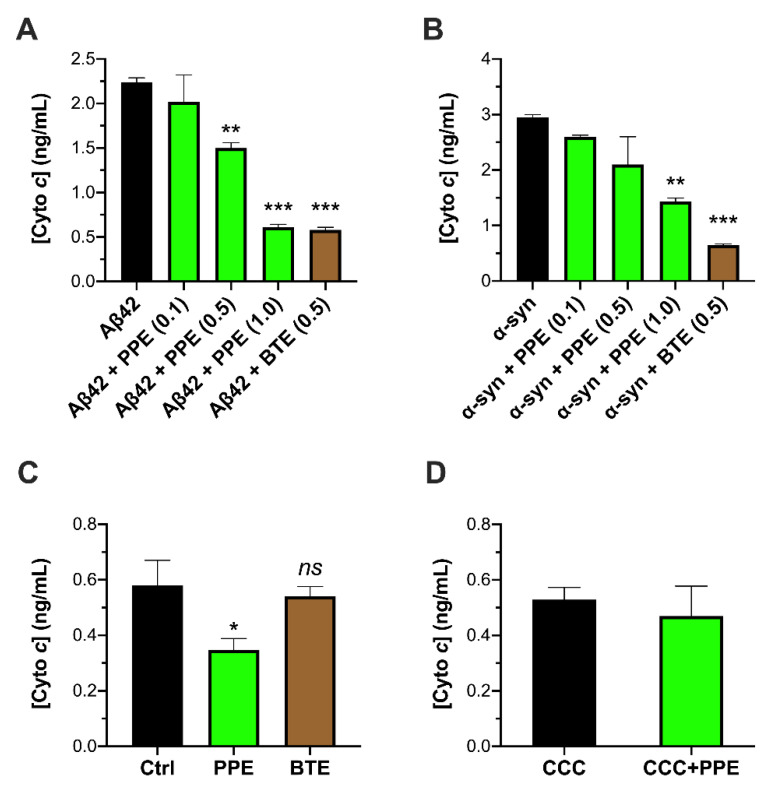
Inhibition of aggregate-induced cyto *c* efflux from isolated mitochondria. (**A**,**B**) Exogenous Aβ42 (5 μM) or α-syn (2 μM) oligomers were added to freshly isolated mitochondria, following a 10-min pre-incubation of the oligomers with 0.01% DMSO (solvent control) alone and the extracts, PPE (0.1–1 μg/mL) or BTE (0.5 μg/mL). The concentration of cyto *c* in the supernatant released from the mitochondria was determined after 30 min in the presence of the Aβ42 or α-syn oligomers. (**C**) Mitochondria were incubated with solvent control (Ctrl; 0.01% DMSO) alone, and with PPE or BTE (in the absence of aggregates). (**D**) To check for possible interference of PPE with the Quantikine^®^ assay, a known concentration of cyto *c* (0.4–0.6 ng/mL) was determined in the absence (CCC) and presence of 1 μg/mL *Padina* extract (CCC+PPE). Values for cyto *c* concentration ([Cyto *c*]) are presented as the means ± standard error of the mean (SEM) performed using duplicate readings (*n* = 3–5). Significance was determined using one-way ANOVA. In (**A**,**B**), *** *p* < 0.001, ** *p* < 0.01, compared to Aβ42 or α-syn alone. In (**C**), *ns* = not significant, * *p* < 0.05, compared to Ctrl. Refer to Suppl. Table S1 for *F*-values and *p*-values of one-way ANOVA.

Previously, we had demonstrated that extract of *Padina pavonica* [27,28] alleviated neurodegenerative phenotypes in fruit fly models of AD and PD when added as a food supplement [32]. In separate studies, we had also shown that pathogenic aggregates of three amyloid-forming proteins involved in the major brain neurodegenerative diseases—Aβ42, α-syn and tau—were highly damaging to mitochondria and the mitochondrial double-membrane. Permeation of mitochondrial membranes was typically manifest with swelling of the organelle and efflux of cyto *c* from the mitochondrial intermembrane space [16,17,33]. Therefore, here we were interested in looking at whether PPE might antagonize aggregate toxicity to mitochondria in vitro. We started by looking at whether PPE can attenuate cyto *c* release (CCR) from isolated mitochondria incubated with aggregated species of Aβ42 and α-syn previously shown to permeabilize lipid membranes [33,34]. Thus, to enable an anti-aggregator effect of the seaweed extract, we allowed PPE to incubate with the pre-formed Aβ42 or α-syn aggregates for 10 min, prior to adding the peptide and PPE mixture (0.1–1 μg/mL) to the mitochondria. We observed that 1 μg/mL PPE exerted a highly significant reduction in both Aβ- and α-syn-induced CCR from mitochondria (Aβ42: from 2.24 ng/mL to 0.61 ng/mL, *p* < 0.001; α-syn: from 2.95 ng/mL to 1.43 ng/mL, *p* < 0.001). The inhibition by PPE was compared to that of black tea extract (BTE) as a ‘positive control’, since BTE is a well-known anti-amyloid and neuroprotectant against both Aβ and α-syn toxicity in vitro and in vivo [35,36,37]. Indeed, PPE was similarly effective to BTE in preventing CCR, especially against Aβ (Figure 1A,B). We also tested PPE and BTE alone with isolated mitochondria, i.e., a 10-min incubation of the extract with mitochondria in the absence of any peptide. Intriguingly, we found that PPE was able to consistently reduce the low ‘background’ CCR that inevitably occurred during the incubation procedure from control mitochondria alone (control: 0.58 ng/mL vs. PPE: 0.35 ng/mL, *p* = 0.0383). This effect was not observed for BTE (BTE: 0.54 ng/mL, *p* > 0.05) (Figure 1C). To exclude any artifactual interference by PPE of the Quantikine^®^ assay, for example by binding to cyto *c* protein, or by non-specific binding to antibodies in the assay, PPE was incubated with a known concentration of cyto *c* protein (provided with the Quantikine^®^ kit) and the assay performed as before. No statistically significant change was found between the cyto *c* concentration as determined in the presence or absence of PPE, thereby establishing that the extract was not itself interfering with the assay (Figure 1D).

**Figure 2:**
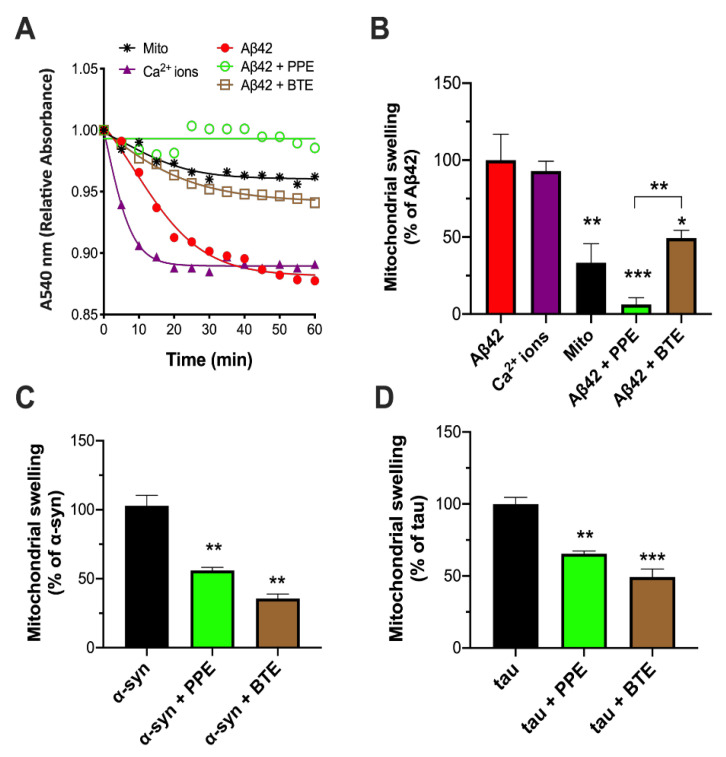
Inhibition of aggregate-induced swelling of isolated mitochondria. Kinetic traces (**A**) of changes in relative absorbance at 540 nm due to swelling of mitochondria upon exposure to 5 μM Aβ42 or 250 μM CaCl_2_ (positive control). *Padina pavonica* extract (PPE; 1 μg/mL), black tea extract (BTE; 0.5 μg/mL) or 0.01% DMSO (solvent control) were incubated with the Aβ42 aggregates at RT for 10 min before adding to the mitochondria. The swelling assays were performed over three independent experiments, representative tracings being shown. Maximal swelling over 1 h in presence of PPE and BTE extracts was calculated as a percentage of that induced by 5 μM Aβ42 (**B**), 2 μM α-syn (**C**) and 1 μM tau (**D**) oligomers alone. Data are presented as means ± SEM (*n* = 3–5); * *p* < 0.05, ** *p* < 0.01, *** *p* < 0.001 relative to protein aggregates alone (one-way ANOVA). Refer to Suppl. Table S1 for *F*-values and *p*-values of one-way ANOVA.

Encouraged by these results, we proceeded to look at changes in mitochondrial volume, since swelling of the mitochondrial matrix is often a precursor to loss of cyto *c* from mitochondria [38]. A convenient and frequently used assay to monitor mitochondrial swelling involves measuring the turbidity in mitochondrial suspensions (A_540_) [38]. Kinetic traces of changes in absorbance were thus obtained for isolated mitochondria exposed to Aβ42 oligomers (Figure 2A), which resulted in a relative decrease in absorbance of −0.119 AU after 1 h. This was similar to the degree of swelling induced by Ca^2+^ ions alone (−0.110 AU, 93 ± 6% of Aβ42 swelling), here used as a positive control since high concentrations of Ca^2+^ ions are known to initiate mitochondrial swelling [39]. Pre-incubation of the Aβ42 aggregates with BTE strongly protected mitochondria from swelling, with a slight increase in mitochondrial volume not significantly different from control mitochondria in respiring buffer alone (control: −0.04 AU, 33 ± 12% of Aβ42 swelling; BTE: −0.06 AU, 49 ± 10% of Aβ42 swelling). The anti-swelling effect of pre-incubating the Aβ42 oligomers with PPE was significantly greater than BTE, with practically no net change in absorbance after 1 h (~0.008 AU, 6 ± 4% of Aβ42 swelling) (Figure 2A,B). It was therefore decided to similarly probe protection by PPE against mitochondrial swelling induced by pre-aggregated α-synuclein and tau proteins. Again, a marked inhibition of swelling (circa 50% less) was found upon addition of PPE to both types of amyloid aggregates, equivalent to inhibition by BTE (Figure 2C,D). As a control, we incubated mitochondria with 1 μg/mL PPE or 0.5 μg/mL BTE for 1 h, but this caused no significant change in absorbance compared to mitochondrial alone (data not shown). Hence, as had been observed in the case of CCR, PPE seemed to exhibit a powerful mito-protectant effect against damage by different oligomers of amyloid proteins.

### 2.2. PPE Protects Mito-Mimetic Membranes from Amyloid Aggregate-Induced Permeabilization and Poration

**Figure 3:**
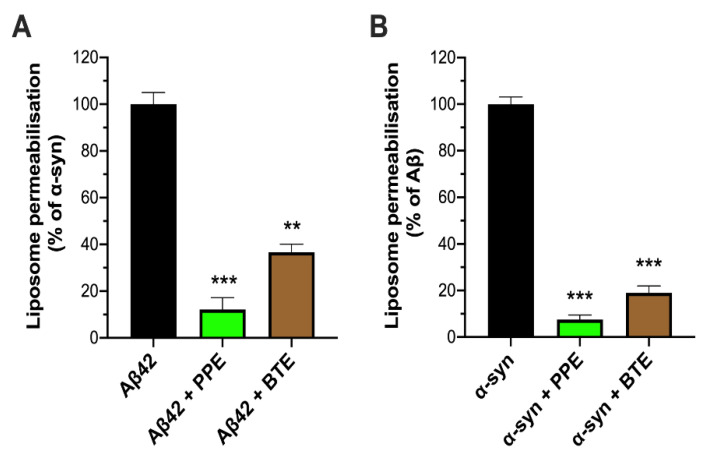
Inhibition of mito-mimetic lipid vesicle permeabilization. *Padina pavonica* extract (PPE; 1 μg/mL), black tea extract (BTE; 1 μg/mL) or 0.01% DMSO (solvent control) were incubated with (**A**) 1 μM Aβ42 or (**B**) 0.5 μM α-syn oligomeric preparation at RT for 10 min, before adding to 60 μM LUVs loaded with the fluorophore Oregon Green^®^. Maximal permeabilization of liposomes in the presence of aggregates was calculated as a percentage of that induced by the aggregates alone (100%). Data are presented as means ± SEM (*n* = 3); ** *p* < 0.01, *** *p* < 0.001 (one-way ANOVA). Refer to Suppl. Table S1 for *F*-values and *p*-values of one-way ANOVA.

We next considered whether the mito-protective effect of PPE could also be demonstrated in minimalist model membranes, consisting of fluorophore-loaded LUVs with a multi-component bilayer mimicking the composition of mitochondrial membranes [33]. Extracts were therefore incubated with pre-aggregated Aβ42 and α-syn for 10 min, before addition to the mito-mimetic liposomes. A powerful inhibitory effect by PPE on peptide-induced permeabilization of the mito-mimetic LUVs was seen: release of encapsulated Oregon Green^®^ fluorophore from the LUVs was substantially reduced to 12% and 8% of that triggered by Aβ42 and α-syn aggregates alone, respectively. This compared favorably, and was in fact slightly better, than the inhibitory effects of BTE (Figure 3). Thus, we were able to show that PPE protects against mitochondrial membrane permeabilization by the amyloid aggregates.

**Figure 4:**
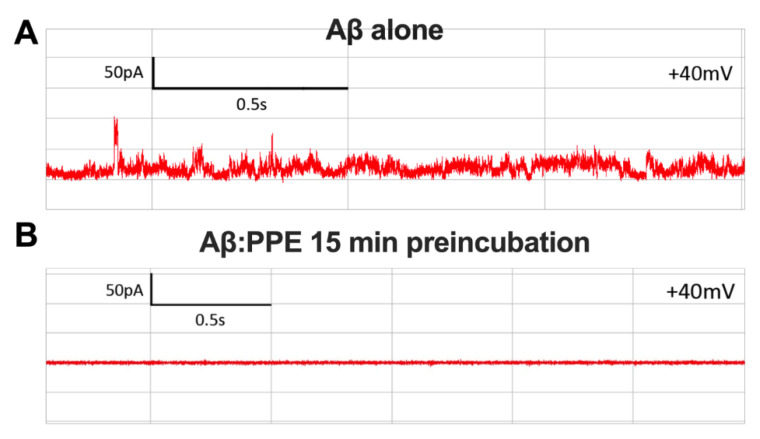
*Padina pavonica* extract prevents amyloid pore electrical activity. Planar lipid bilayer (45 PC/25 PE/10 PI/5 PS/15 CL) electrical recordings showed multilevel conductance events when oligomeric preparations of 0.5 μM Aβ42, 1 μM α-syn and 0.4 μM tau were exogenously added to the test chamber—a representative trace of Aβ42 amyloid pore current activity is illustrated in (**A**). Remarkably, a 15-min preincubation of the oligomers with PPE (1 μg/mL) prior to membrane introduction completely prevented ionic flux across the mito-mimetic planar bilayer for 2+ h of recording (**B**). Symmetrical KCl buffer (250 mM) solutions were used in the *cis* and *trans* chambers, with voltage-clamping at +40 mV.

In previous work, we had shown that permeabilization of mitochondrial-like bilayers by amyloid oligomers of α-syn and tau is associated with the formation of large and stable nanopores in the membrane that allow ionic flux [16,17]. Therefore, we next interrogated whether PPE could inhibit formation of amyloid nanopores by Aβ42, α-syn and tau oligomers in a single-channel electrophysiology setup, using an identical mito-mimetic composition for the planar bilayer as in the liposome assays. Prior to introduction to the test chamber, oligomeric preparations of Aβ42, α-syn and tau were pre-incubated with PPE for 15 min. Pore activity was monitored in the tracings of ionic current passing through the planar lipid bilayer (Figure 4A). 

Electrical recordings characteristic of pore formation were completely absent when either of the three types of protein oligomers had been pre-incubated with PPE (0 out of 6 trials each for Aβ42, α-syn and tau protein), with no deviation of the current tracings from baseline detected for at least 2 h of recording (Figure 4B). This in comparison to a rate of pore formation of 45–75% when the oligomers alone were added to the *cis*-chamber (*n* = 6 trials for each peptide/protein) [16,17]. Given the laborious (low-throughput) nature of the electrophysiological method, BTE was tested against α-syn oligomers only. It was found to be marginally less effective than PPE, with a pore formation frequency of 17% (1 out of 6 trials), compared to 67% (4 out of 6 trials) with α-syn oligomers alone.

### 2.3. PPE Modulates the Fibrillization Pathways of Aβ42 and α-Synuclein 

**Figure 5:**
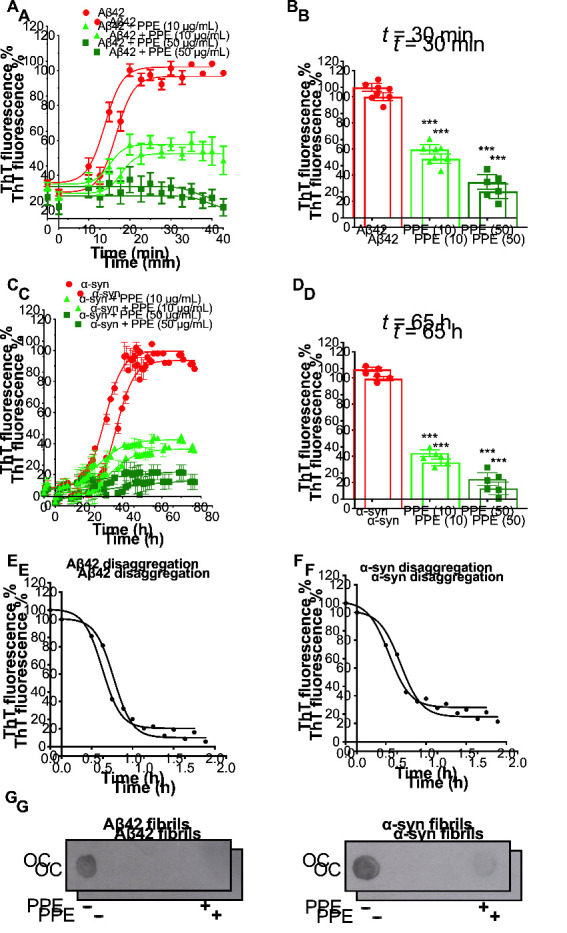
Effects of *Padina pavonica* extract on fibrillogenesis of Aβ42 and α-syn. (**A**,**C**) Aggregation kinetics of 22.5 μM Aβ42 and 25 μM α-syn were monitored using Thioflavin T, with and without PPE (10 or 50 μg/mL) in phosphate buffer saline (PBS) buffer. Aggregations were performed at 37 °C with agitation at 450 rpm. ThT fluorescence readings were measured in triplicate, and the means ± SD are shown at each data point, calculated as a percentage of the maximal ThT fluorescence intensities of the control peptide/protein aggregated alone (*n* = 3). The kinetic data was fitted to a sigmoidal curve using GraphPad Prism. (**B**,**D**) Bar diagram showing maximal ThT signals achieved in the plateau phase of aggregation (at *t* = 30 min for Aβ42 and *t* = 65 h for α-syn), in the absence and presence of PPE (10 or 50 μg/mL) as a percentage of control peptide/protein aggregated alone. Values are expressed as means ± SD (*n* = 3); *** *p* < 0.001 (one-way ANOVA). Refer to Suppl. Table S1 for *F*-values and *p*-values of one-way ANOVA. (**E**,**F**) Disaggregation properties of PPE are illustrated by time-dependent ThT fluorescence profiles following addition of PPE (10 μg/mL) to completely aggregated Aβ42 or α-syn (regarded as 100%). Data points represent the means ± SD of three replicate experiments (*n* = 3) expressed as percentages of the control. (**G**) Dot blots were probed with Fibril OC antibody to detect Aβ42 (*left panel*) and α-syn (*right panel*) fibrils, prepared in the absence (−) or after the addition of PPE (10 μg/mL) for 2 h (+). A fainter spot indicates that amyloid fibrils were significantly reduced by PPE.

Thioflavin T (ThT) is a widely used fluorescent dye to kinetically monitor the formation of amyloid fibrils along the aggregation pathway [40]. Upon binding within the cross-β-architecture along the long axis of amyloid fibrils, ThT emits a strong fluorescence signal [41]. Previous studies have shown prevention of aggregation and disaggregation of fibril formation of the amyloid-β peptide fragment Aβ(25–35) by extracts of the seaweed *P. gymnospora* [30]. We therefore sought to determine the effects of PPE on the fibrillization pathways of Aβ42 and α-syn using the ThT binding assay. In the absence of PPE, both Aβ42 and α-syn fully aggregated into β-sheet-rich amyloid fibrils incorporating ThT molecules: in line with published literature, the highly amyloidogenic amyloid-β peptide took less than 1 h, while the α-syn protein took ~2 days to reach maximal ThT fluorescence under the experimental conditions used [40,42]. In the presence of PPE, however, formation of Aβ42 and α-syn amyloid fibrils was powerfully suppressed (Figure 5A,C). At 10 μg/mL PPE, a much slower rate of fibril growth was observed, with ThT intensity reaching 52 ± 4% for Aβ42 and 35 ± 2% for α-syn ThT-positive fibrils. At a higher 50 μg/mL PPE concentration, minimal fibril formation occurred for the duration of the experiment, with peak ThT fluorescence only reaching 27 ± 6% of Aβ42 and 15 ± 5% of α-syn alone (Figure 5B,D). The above experiments thus indicate that PPE exhibits excellent activity against the polymerization of Aβ42 and α-syn monomers into fibrillary aggregates.

In view of the fact that in our experiments we always incubated pre-aggregated Aβ42 or α-syn with PPE, we decided to additionally test the amyloid-disrupting properties of the seaweed extract using the ThT assay. Therefore, PPE (10 μg/mL) was added to pre-formed fibrils of Aβ42 and α-syn, and the ThT fluorescence intensity tracked for up to 2 h. The ThT signal for amyloid fibrils incubated with PPE decreased prominently following an inverse sigmoidal curve, to 10% and 21% for Aβ42 and α-syn fibrils, respectively, suggesting extensive disruption of the fibrillar β-architecture by PPE (Figure 5E,F). Another method, that of immunoblotting using an antibody which specifically binds to the fibrillary form of Aβ42 and α-syn, was used to more directly visualize the disaggregation potential of PPE. In accordance with the ThT assays, very faint spots were seen after incubation of the fibrils with PPE, indicating a highly effective disaggregation activity (Figure 5G).

Thus, the results of the antiaggregation and disaggregation assays together indicate that PPE curtails the ability of two key amyloid disease proteins, Aβ42 and α-syn, to self-associate and accrue into larger β-aggregate structures, as well as potently disrupt the respective mature fibrils.

## 3. Discussion

Defects in neuronal mitochondria are linked to early pathophysiology of several human neurodegenerative disorders of the amyloid type, such as AD and PD [43]. In such disorders, mitochondrial dysfunction in neurons and synapses can be triggered by toxic conformations of intrinsically disordered proteins such as Aβ42, α-syn and tau, directly interacting with mitochondria to cause mitochondrial poration, increased membrane permeability and swelling, and through dysfunctional OXPHOS, diminished ATP production [16,17,44,45]. Indeed, neuronal and synaptic mitochondria are especially more susceptible to damage and more sensitive to swelling than non-neuronal mitochondria [46]. Finding therapeutic molecules that help preserve and restore mitochondrial integrity in the face of onslaught by neurotoxic amyloid entities, thus represents an important goal in the search for effective treatment of these neurodegenerative diseases.

Our findings indicate that an acetonic extract derived from the marine brown seaweed *Padina pavonica* demonstrates remarkable mito-protective properties, by preserving the membrane integrity of mitochondria directly exposed to membrane-active aggregates of three amyloid proteins (Aβ42, α-syn and tau) believed to play important causal roles in the most common neurodegenerative diseases [16,17,47]. Specifically, a robust (~50% or more) decrease in abnormal morphology (swelling) and in cyto *c* efflux from isolated mitochondria were observed, after pre-incubation of the amyloid oligomers with PPE. The mito-protective effects of PPE were especially prominent against the Aβ42 aggregates. Interestingly, both mitochondrial swelling and loss of cyto *c* from mitochondria have been shown using multiphoton microscopy in the brains of living mouse models of AD (APP/PS1 transgenic mice) in the vicinity of Aβ plaques [48]. Similarly, α-syn overexpression in mouse brain resulted in enlarged and swollen mitochondria, as well as increased levels of cyto *c* in the cytosol [49]. Hence, our in vitro model using isolated mitochondria from SH-SY5Y cells and low micromolar concentrations of aggregated recombinant synthetic peptides, in which we showed inhibitory activity of PPE, recapitulated two prominent mitochondrial events seen in amyloid mouse models of AD and PD. Furthermore, our inference that PPE is effectively preventing disruption of mitochondrial membranes is reinforced by the finding that oligomeric aggregates exposed to PPE were much less able to permeabilize mitomimetic LUVs, or form ion-conducting nanopores in the bilayer membrane (BLM). Formation of pore-like structures in the mitochondrial outer membrane were seen in atomic force microscopy (AFM) images of mitochondria from brains of transgenic mice exhibiting expression of human α-syn [49]. Therefore, although our minimal reconstituted model membrane systems oversimplify the true complexity of mitochondrial membranes, they can nonetheless provide a powerful experimental means to obtain mechanistic insights [50,51].

One aspect we wanted to explore further was the anti-aggregation activity of PPE. In the present study, experiments involving PPE were conducted after first pre-incubating the amyloid aggregates with the extract. Hence, it would be reasonable to assume that during this time, molecular constituents present in the extract interacted with the membrano-toxic amyloid entities and converted them into less harmful aggregates. In agreement with this notion, PPE manifested potent disaggregating activity of the β-sheet structure of Aβ42 and α-syn fibrillary aggregates, which possess an otherwise highly stable antiparallel β-architecture [52]. The anti-aggregation properties of PPE were further substantiated by demonstrating strong inhibition of Aβ42 and α-syn amyloidogenesis in the ThT-based assays. The latter kinetic assays complement immunoblotting experiments carried out previously by our group showing that PPE suppressed the formation of both Aβ42 and α-syn protofibrils [32]. In support of our work, acetone extracts of another *Padina* species, *Padina gymnospora*, prevented aggregation and caused disaggregation of mature fibrils of the Aβ25–35 peptide, which in an aggregated state is toxic to cultured neurons [30]. Chemical composition studies of the acetone extract used in the present study identified a rich content of polyphenols, in particular flavonoids, and tannins. The role of polyphenols on the aggregation and disaggregation of Aβ peptide, tau and α-syn haven been extensively studied and described [53]. Naturally occurring dietary flavonoids have in fact gained considerable attention as providing an alternative approach to slowing the progression of AD or PD pathogenesis [54]. Possible mechanisms include modulation of monomer-monomer interactions, inhibition of oligomerization into a toxic species, and remodeling of toxic confomers into nontoxic forms by way of hydrogen bonding, electrostatic effects and/or π-π (pi-pi) stacking [55,56].

Although it is indeed most likely that the principal mechanism of action of PPE is via association with, and modification of, the toxic oligomeric structures, we cannot exclude a priori a concomitant modulation of the permeability of the phospholipid membrane by the seaweed extract. In this regards, it is pertinent to draw attention to the apparent mito-protective effect of PPE on isolated mitochondria alone, in which the background release of cyto *c* from mitochondria incubated with PPE was significantly lower than from control mitochondria: this protective effect was robust and specific to PPE. Thus, we may speculate that the phenols, sterols or terpenoids found in *Padina pavonica* extract could possibly be altering physicochemical properties of the mitochondrial membranes, such as their membrane fluidity [57,58]. Further experimental work is underway in order to delve deeper into this phenomenon, which might have important implications on the biological activity of PPE.

Another interesting point to come out from this work is that in the mitochondrial and liposome permeabilization assays, as well as in the electrical recordings for amyloid pores, PPE was as effective, and in several instances even more effective, than a theaflavin-based extract from black tea in its anti-amyloid activity. Notably, BTE and theaflavins, the main polyphenolic components found in fermented black tea, were reported as among the strongest inhibitors of Aβ42 and α-syn fibrillogenesis [34,56,59]. Hence, even more than BTE, PPE is a potent dual inhibitor of both Aβ42 and α-syn toxicity—few amyloid inhibitors have been found to have excellent activities against different amyloid peptides, and this challenge has become even more pressing given the multiple reports of co-assembly and co-deposition of amyloidogenic peptides in vitro and in vivo into hetero-amyloids [60,61,62]. As with other herbal extracts, it is likely that the extract milieu of PPE may be crucial for providing such optimal bioactivity. In this manner, synergies among the multiple single components of the extract may provide an ideal environment in which the effect of the natural product mixture is greater than that of the individual purified compounds [63,64]. Further studies are therefore underway to explore the biological effects of the whole PPE extract formula on the complex disease-related molecular network represented by amyloid pathology.

## 4. Materials and Methods

### 4.1. Padina Pavonica and Black Tea Extracts

Extract derived from the alga *Padina pavonica* (PPE) was supplied by the Institute of Cellular Pharmacology (ICP Concepts Ltd., Mosta Technopark, Malta). PPE was produced and chemically characterized as described previously [27,28,65]. Briefly, the seaweed was dried and milled before solid-liquid extraction was carried out by the Soxhlet extraction method using acetone as solvent. The extracted product was then filtered and fed into a rotary evaporator where it was dried under vacuum at 55 °C for several hours. PPE was supplied in the form of crude extract of the active fraction. Stocks (10 mg/mL) in 100% dimethyl sulfoxide (DMSO) were stored at −20 °C.

Black tea extract (BTE; >80% theaflavins) was obtained from Sigma-Aldrich (Munich, Germany). Stocks (10 mg/mL) in 100% DMSO were stored at −20 °C.

### 4.2. Preparation of Amyloid Aggregates

Oligomer-rich preparations of aggregates of Aβ42 (rPeptide, Ely, UK), human recombinant α-syn ([17]) and human recombinant tau protein (hTau46, 1N4R isoform [16]) were prepared according to previously established protocols. Briefly, 45 μM monomeric Aβ was incubated for 2 h in sterile phosphate buffered saline (PBS, pH 7.4) at 37 °C without shaking [34]; 7 μM monomeric αS was incubated for 72 h in 50 mM Tris-HCl (pH 7.0) with 1% DMSO and 20 μM FeCl_3_ without agitation at 25 °C [66]; 7 μM monomeric tau was incubated in 70 μM AlCl_3_ and Dulbecco’s PBS (DPBS) without agitation at 37 °C for 96 h [67]. To avoid repeated freeze/thawing, aggregated samples were divided into aliquots in LoBind tubes (Eppendorf, Hamburg, Germany) before storing at −80 °C.

### 4.3. Thioflavin T (ThT) Fluorescence Fibril Assay

To detect the formation of Aβ and α-syn fibrils, ThT (final concentration 40 μM in PBS, pH 7.4) was added to wells in a black and clear flat bottom, non-binding microplate (Corning^®^ catalog number 3881, New York, NY, USA), and mixed with aggregated protein (Aβ42 or α-syn) alone and in the presence of *Padina pavonica* extract (10–50 μg/mL). The plate was sealed with clear polyolefin tape and incubated at 37 °C with agitation at 450 rpm. Fluorescence intensities of the solutions were subsequently measured using a FLx800 microplate reader (Bio-Tek, Bedfordshire, UK) with excitation and emission wavelengths at 445 nm and 490 nm, respectively. Fluorescence readings were background subtracted by that of ThT alone. For the disaggregation assay, 10 μg/mL *Padina pavonica* extract was added to preformed Aβ42 or α-syn fibrils (22.5 μM Aβ42 for 1 h; 25 μM α-syn for 72 h) and mixed thoroughly. Then, ThT was added and fluorescence of the solution was measured at 37 °C without shaking for 100 min.

### 4.4. Preparation of Isolated Mitochondria from SH-SY5Y Cells

Isolated mitochondria for the cyto *c* assay and swelling experiments were prepared fresh for each experiment from ~5 × 10^7^ cultured SH-SY5Y human neuroblastoma cells (ATCC^®^ CRL-2266TM, Manassas, VA, USA) using the MITOISO2^®^ kit (Sigma-Aldrich, Germany) according the manufacturer’s instructions. For downstream application, the mitochondrial pellet was resuspended in respiring buffer (50 mM HEPES, pH 7.5, containing 1.25 M sucrose, 25 mM succinate, 5 mM ATP, 0.4 mM ADP, 10 mM K_2_HPO_4_) at ~1–2 mg/mL (final mitochondrial protein concentration determined using NanoOrange^®^ kit, ThermoFisher Scientific, Waltham, MA, USA). Mitochondria were kept on ice during the entire isolation procedure. Purity of the “heavy” mitochondrial fraction was confirmed as described previously [33].

### 4.5. Quantikine^®^ Immunoassay for Determination of Cytochrome c Release

The Quantikine^®^ assay kit (R&D Systems, Ely, UK) provides for an accurate quantification of cytochrome *c* in supernatant fractions using a colorimetric ELISA method [33]. Thus, fresh isolated mitochondria (~12 μg) in respiring buffer were incubated for 30 min at 37 °C, alone or in the presence of pre-aggregated amyloid oligomers; when needed, the pre-formed oligomers were left for 10 min in presence of extract (PPE or BTE) at room temperature prior to the addition to mitochondria. Following centrifugation (16,000× *g* for 10 min, 4 °C), the supernatant was used for the cyto *c* immunoassay as per kit instructions. Background CCR from control mitochondria not exposed to peptides was subtracted from other values.

### 4.6. Mitochondrial Swelling Assays

Mitochondrial swelling was determined by measuring changes in mitochondrial volume as described [16]. Briefly, mitochondria (1–2 mg/mL of protein) were incubated in 80 μL of respiration buffer containing 10 mM HEPES, 5 mM succinate, 250 mM sucrose, 1 mM ATP, 0.08 mM ADP, 2 mM K_2_HPO_4_, pH 7.5 at 25 °C. Baseline levels of absorbance at 540 nm (OD ~0.35–0.40) were measured for 10 min to ensure stability of mitochondria, and the optical density monitored for 60 min after the addition of oligomeric peptide (Aβ42, α-syn, or tau). Where needed, extracts were incubated with the protein aggregates for 10 min before being added to the mitochondria. 

### 4.7. Preparation of Mito-Mimetic Liposomes and Vesicle Leakage Assays

Lipids in chloroform were all purchased from Avanti Polar Lipids (Alabaster, AL, USA). Briefly, the lipids were mixed at the following ratios (by % weight): 45 PC (phosphatidylcholine), 25 PE (phosphatidylthethanolamine), 10 PI (phosphatidylinositol), 5 PS (phosphatidylserine), 15 CL (caradiolipin), which mimics the composition of the outer mitochondrial contact sites and the inner mitochondrial membrane [68,69]. Large unilamellar vesicles (LUVs) loaded with Oregon Green^®^ 488 BAPTA-1 fluorophore (OG; ThermoFisher Scientific, Waltham, MA, USA) were prepared using the detergent-dialysis method as described previously [33]. The size and uniformity of the vesicle population were checked using a Zetasizer Nano S dynamic light scattering (DLS) device (Malvern, Worcestershire, UK). The vesicles were relatively uniformly sized with an average diameter of 87 ± 20 nm, and hence categorized as LUVs. 

Permeabilization assays were carried out as described [33]. Briefly, protein aggregate preparations were added to 60 μM lipid vesicles in liposome buffer (100 mM KCl, 10 mM MOPS/Tris, 1 mM EDTA, 0.1 mM CaCl_2_, pH 7.0) and fluorescence readings kinetically monitored for 60 min using a FLx800 (BioTek, Winooski, VT, USA) microplate reader (exc. 485 nm, em. 528 nm). Disruption of lipid vesicles by aggregates in the presence of extracts (BTE or PPE) was calculated as a percentage of permeabilization caused by aggregates alone (theoretical maximum, 100%). 

### 4.8. Planar Lipid Bilayer Electrophysiology

Ion current across planar lipid bilayers was recorded using single-channel electrical recordings on an Ionovation Compact automated bilayer workstation (Ionovation GmbH, Osnabrück, Germany) as described previously [17,70]. Mito-mimetic bilayers were formed using the same defined lipid ratios as for the LUV preparation (45 PC/25 PE/10 PI/5 PS/15 CL) by spreading the lipid up-and-down (“painting technique”) across a ~120 μm aperture in a Teflon septum (Ionovation GmbH, Osnabrück, Germany) separating *cis* and *trans* compartments containing electrolyte (250 mM KCl, 10 mM MOPS/Tris, pH 7.2). Formation of the bilayer membrane was verified throughout the experiment, visually using a built-in low amplification microscope and by taking capacitance measurements. In previous studies, this membrane composition was shown to be stable for at least 2 h (typical capacitance of 50–70 pF and a conductance of 12–14 pS) [16,17]. Oligomeric peptide preparations of Aβ42, α-syn or tau were added to the electrically grounded *cis*-chamber just below the bilayer. Experiments were performed with peptide aliquots that had not been freeze-thawed more than once. Chambers contained magnetic stirrers to facilitate oligomer incorporation into the bilayer. To evaluate the effect of extracts on amyloid pore formation, the aggregate preparation was preincubated for 15 min with 1 μg/mL PPE or 0.5 μg/mL BTE before introducing into the electrolyte solution. Preliminary experiments had determined that at these concentrations the extracts caused no increase in ionic current over baseline over at least 4 h of recording (*n* = 3 for each extract). Measurements of transmembrane currents were recorded in applied ±40 mV voltage clamp mode using a HEKA^®^ EPC10 amplifier with a sampling frequency of 15 kHz. Data acquisition was carried out using Patchmaster software version 2x90 (HEKA, Lambrecht/Pfalz, Germany).

### 4.9. Dot Blot Assay 

Dot blot assays were performed using the fibril-specific OC antibody [71]. Briefly, samples of 4 μL containing Aβ or α-syn were spotted onto a nitrocellulose membrane (Hybond-ECL, GE Life Sciences) and after air-drying, membranes were blocked with 2.5% BSA in Tris-buffered saline containing 0.1% (*v*/*v*) Tween-20 (TBS-T) for 1 h at room temperature. After rinsing briefly with TBS, membranes were probed with the OC antibody (1:2000 in TBS; AB2286, Millipore, Bedford, MA, USA) for 2 h at room temperature. The membranes were then washed three times for 5 min each with TBS-T on an orbital shaker, and incubated with secondary horseradish peroxidase-conjugated anti-rabbit antibody (1:5000 in TBST) for 1 h at room temperature. Three subsequent washes were performed with TBS-T and the last wash with TBS only, for 5 min each. Lastly, the blots were developed using the ECL immunoblotting kit (RPN2108, GE Life Sciences, Little Chalfont, United Kingdom) as per manufacturer instructions.

### 4.10. Statistical Analysis

All statistical analyses were performed using GraphPad Prism v8 (GraphPad Software, San Diego, CA, USA). Statistical significance was examined by one-way ANOVA and Bonferroni’s multiple comparisons tests (*F*-values and *p*-values of one-way ANOVA are provided in Suppl. Table S1). Normality was assessed on all samples subjected to statistical analysis to ensure data met the assumptions of the tests used and statistical outliers identified. The data are presented as means ± standard error of the mean (SEM) unless stated otherwise, with *n* as the number of independent experiments.

## 5. Conclusions

Extracts from seaweed plants and their bioactive compounds are becoming increasingly recognized as useful resources of molecules to help combat neurological disease of the amyloid type. An effective treatment will likely include a combination of drugs that protect mitochondrial function and prevent amyloid accumulation. Here we showed that *Padina pavonica* extract is an efficient anti-aggregator of amyloid proteins and protects mitochondria organelles by preserving mitochondrial membrane integrity. Further investigations using cellular models of AD and PD, characteristically involving overexpression of the wild-type or mutant amyloid protein, will be required to evaluate further whether the protection of mitochondria by *Padina pavonica* extract represents a potential route to combat the pathological effects associated with aggregation in neurodegenerative proteinopathies.

## Data Availability

The data used to support the findings of this study are available from the corresponding author upon request.

## Supplementary Materials

The following are available online, Table S1: *F*-values and *p*-values pertaining to one-way ANOVA.

## Sample Availability

Not available.

## References

[1] Chiti F., Dobson C.M. (2017). Protein Misfolding, Amyloid Formation, and Human Disease: A Summary of Progress Over the Last Decade. Annu. Rev. Biochem..

[2] Soto C., Estrada L.D. (2008). Protein misfolding and neurodegeneration. Arch. Neurol..

[3] Knowles T.P., Vendruscolo M., Dobson C.M. (2014). The amyloid state and its association with protein misfolding diseases. Nat. Rev. Mol. Cell Biol..

[4] Breydo L., Uversky V.N. (2015). Structural, morphological, and functional diversity of amyloid oligomers. FEBS Lett..

[5] Mannini B., Mulvihill E., Sgromo C., Cascella R., Khodarahmi R., Ramazzotti M., Dobson C.M., Cecchi C., Chiti F. (2014). Toxicity of protein oligomers is rationalized by a function combining size and surface hydrophobicity. ACS Chem. Biol..

[6] Diociaiuti M., Macchia G., Paradisi S., Frank C., Camerini S., Chistolini P., Gaudiano M.C., Petrucci T.C., Malchiodi-Albedi F. (2014). Native metastable prefibrillar oligomers are the most neurotoxic species among amyloid aggregates. Biochim. Biophys. Acta..

[7] Olzscha H., Schermann S.M., Woerner A.C., Pinkert S., Hecht M.H., Tartaglia G.G., Vendruscolo M., Hayer-Hartl M., Hartl F.U., Vabulas R.M. (2011). Amyloid-like aggregates sequester numerous metastable proteins with essential cellular functions. Cell.

[8] Angleova P.R., Ludtmann M.H., Horrocks M.H., Negoda A., Cremades N., Klenerman D., Dobson C.M., Wood N.W., Pavlov E.V., Gandhi S. (2016). Ca^2+^ is a key factor in alpha-synuclein-induced neurotoxicity. J. Cell Sci..

[9] Campioni S., Mannini B., Zampagni M., Pensalfini A., Parrini C., Evangleisti E., Relini A., Stefani M., Dobson C.M., Cecchi C. (2010). A causative link between the structure of aberrant protein oligomers and their toxicity. Nat. Chem. Biol..

[10] Shrivastava A.N., Aperia A., Melki R., Triller A. (2017). Physico-Pathologic Mechanisms Involved in Neurodegeneration: Misfolded Protein-Plasma Membrane Interactions. Neuron.

[11] Butterfield S.M., Lashuel H.A. (2010). Amyloidogenic protein-membrane interactions: Mechanistic insight from model systems. Angew. Chem. Int. Ed. Engl..

[12] Kagan B.L. (2012). Membrane pores in the pathogenesis of neurodegenerative disease. Prog. Mol. Biol. Transl. Sci..

[13] Fusco G., Chen S.W., Williamson P.T.F., Cascella R., Perni M., Jarvis J.A., Cecchi C., Vendruscolo M., Chiti F., Cremades N. (2017). Structural basis of membrane disruption and cellular toxicity by alpha-synuclein oligomers. Science.

[14] Farrugia M.Y., Caruana M., Ghio S., Camilleri A., Farrugia C., Cauchi R.J., Cappelli S., Chiti F., Vassallo N. (2020). Toxic oligomers of the amyloidogenic HypF-N protein form pores in mitochondrial membranes. Sci. Rep..

[15] Todorova V., Blokland A. (2017). Mitochondria and Synaptic Plasticity in the Mature and Aging Nervous System. Curr. Neuropharmacol..

[16] Camilleri A., Ghio S., Caruana M., Weckbecker D., Schmidt F., Kamp F., Leonov A., Ryazanov S., Griesinger C., Giese A. (2020). Tau-induced mitochondrial membrane perturbation is dependent upon cardiolipin. Biochim. Biophys. Acta. Biomembr..

[17] Ghio S., Camilleri A., Caruana M., Ruf V.C., Schmidt F., Leonov A., Ryazanov S., Griesinger C., Cauchi R.J., Kamp F. (2019). Cardiolipin Promotes Pore-Forming Activity of Alpha-Synuclein Oligomers in Mitochondrial Membranes. ACS Chem. Neurosci..

[18] Vicario M., Cieri D., Brini M., Cali T. (2018). The Close Encounter Between Alpha-Synuclein and Mitochondria. Front Neurosci..

[19] Shafiei S.S., Guerrero-Munoz M.J., Castillo-Carranza D.L. (2017). Tau Oligomers: Cytotoxicity, Propagation, and Mitochondrial Damage. Front Aging. Neurosci..

[20] Cha M.Y., Han S.H., Son S.M., Hong H.S., Choi Y.J., Byun J., Mook-Jung I. (2012). Mitochondria-specific accumulation of amyloid beta induces mitochondrial dysfunction leading to apoptotic cell death. PLoS ONE.

[21] McInnes J. (2013). Insights on altered mitochondrial function and dynamics in the pathogenesis of neurodegeneration. Transl. Neurodegener..

[22] Price K.A., Varghese M., Sowa A., Yuk F., Brautigam H., Ehrlich M.E., Dickstein D.L. (2014). Altered synaptic structure in the hippocampus in a mouse model of Alzheimer’s disease with soluble amyloid-beta oligomers and no plaque pathology. Mol. Neurodegener..

[23] Orlando-Bonaca M., Lipej L., Orfanidis S. (2008). Benthic macrophytes as a tool for delineating, monitoring and assessing ecological status: The case of Slovenian coastal waters. Mar. Pollut. Bull.

[24] Ngo D.H., Vo T.S., Ngo D.N., Wijesekara I., Kim S.K. (2012). Biological activities and potential health benefits of bioactive peptides derived from marine organisms. Int. J. Biol. Macromol..

[25] Admassu H., Gasmalla M.A.A., Yang R., Zhao W. (2018). Bioactive Peptides Derived from Seaweed Protein and Their Health Benefits: Antihypertensive, Antioxidant, and Antidiabetic Properties. J. Food Sci..

[26] Behmer S.T., Olszewski N., Sebastiani J., Palka S., Sparacino G., Sciarrno E., Grebenok R.J. (2013). Plant phloem sterol content: Forms, putative functions, and implications for phloem-feeding insects. Front Plant. Sci..

[27] Bernardini G., Minetti M., Polizzotto G., Biazzo M., Santucci A. (2018). Pro-Apoptotic Activity of French Polynesian Padina pavonica Extract on Human Osteosarcoma Cells. Mar. Drugs.

[28] Minetti M., Bernardini G., Biazzo M., Gutierrez G., Geminiani M., Petrucci T., Santucci A. (2019). Padina pavonica Extract Promotes In Vitro Differentiation and Functionality of Human Primary Osteoblasts. Mar. Drugs.

[29] Schepers M., Martens N., Tiane A., Vanbrabant K., Liu H.B., Lutjohann D., Mulder M., Vanmierlo T. (2020). Edible seaweed-derived constituents: An undisclosed source of neuroprotective compounds. Neural. Regen. Res..

[30] Shanmuganathan B., Sheeja Malar D., Sathya S., Pandima Devi K. (2015). Antiaggregation Potential of Padina gymnospora against the Toxic Alzheimer’s Beta-Amyloid Peptide 25–35 and Cholinesterase Inhibitory Property of Its Bioactive Compounds. PLoS ONE.

[31] Shanmuganathan B., Sathya S., Balasubramaniam B., Balamurugan K., Devi K.P. (2019). Amyloid-beta induced neuropathological actions are suppressed by Padina gymnospora (Phaeophyceae) and its active constituent alpha-bisabolol in Neuro2a cells and transgenic Caenorhabditis elegans Alzheimer’s model. Nitric. Oxide.

[32] Briffa M., Ghio S., Neuner J., Gauci A.J., Cacciottolo R., Marchal C., Caruana M., Cullin C., Vassallo N., Cauchi R.J. (2017). Extracts from two ubiquitous Mediterranean plants ameliorate cellular and animal models of neurodegenerative proteinopathies. Neurosci. Lett..

[33] Camilleri A., Zarb C., Caruana M., Ostermeier U., Ghio S., Hogen T., Schmidt F., Giese A., Vassallo N. (2013). Mitochondrial membrane permeabilisation by amyloid aggregates and protection by polyphenols. Biochim. Biophys. Acta..

[34] Gauci A.J., Caruana M., Giese A., Scerri C., Vassallo N. (2011). Identification of polyphenolic compounds and black tea extract as potent inhibitors of lipid membrane destabilization by Abeta(4)(2) aggregates. J. Alzheimers Dis..

[35] Li X., Smid S.D., Lin J., Gong Z., Chen S., You F., Zhang Y., Hao Z., Lin H., Yu X. (2019). Neuroprotective and Anti-Amyloid beta Effect and Main Chemical Profiles of White Tea: Comparison Against Green, Oolong and Black Tea. Molecules.

[36] Anandhan A., Tamilselvam K., Radhiga T., Rao S., Essa M.M., Manivasagam T. (2012). Theaflavin, a black tea polyphenol, protects nigral dopaminergic neurons against chronic MPTP/probenecid induced Parkinson’s disease. Brain Res..

[37] Caruana M., Vassallo N. (2015). Tea Polyphenols in Parkinson’s Disease. Adv. Exp. Med. Biol..

[38] Karch J., Kwong J.Q., Burr A.R., Sargent M.A., Elrod J.W., Peixoto P.M., Martinez-Caballero S., Osinska H., Cheng E.H., Robbins J. (2013). Bax and Bak function as the outer membrane component of the mitochondrial permeability pore in regulating necrotic cell death in mice. Elife.

[39] Ichimura T., Ito M., Takahashi K., Oyama K., Sakurai K. (2011). Involvement of mitochondrial swelling in cytochrome *c* release from mitochondria treated with calcium and Alloxan. J. Biophys. Chem..

[40] Xue C., Lin T.Y., Chang D., Guo Z. (2017). Thioflavin T as an amyloid dye: Fibril quantification, optimal concentration and effect on aggregation. R. Soc. Open Sci..

[41] Biancalana M., Koide S. (2010). Molecular mechanism of Thioflavin-T binding to amyloid fibrils. Biochim. Biophys. Acta..

[42] Wordehoff M.M., Hoyer W. (2018). alpha-Synuclein Aggregation Monitored by Thioflavin T Fluorescence Assay. Bio. Protoc..

[43] Wu Y., Chen M., Jiang J. (2019). Mitochondrial dysfunction in neurodegenerative diseases and drug targets via apoptotic signaling. Mitochondrion.

[44] Pantiya P., Thonusin C., Chattipakorn N., Chattipakorn S.C. (2020). Mitochondrial abnormalities in neurodegenerative models and possible interventions: Focus on Alzheimer’s disease, Parkinson’s disease, Huntington’s disease. Mitochondrion.

[45] Grimm A., Eckert A. (2017). Brain aging and neurodegeneration: From a mitochondrial point of view. J. Neurochem..

[46] Olesen M.A., Torres A.K., Jara C., Murphy M.P., Tapia-Rojas C. (2020). Premature synaptic mitochondrial dysfunction in the hippocampus during aging contributes to memory loss. Redox. Biol..

[47] Walker L., Attems J. (2019). Relationship Between Tau, beta Amyloid and alpha-Synuclein Pathologies. Adv. Exp. Med. Biol..

[48] Xie H., Guan J., Borrelli L.A., Xu J., Serrano-Pozo A., Bacskai B.J. (2013). Mitochondrial alterations near amyloid plaques in an Alzheimer’s disease mouse model. J. Neurosci..

[49] Gao G., Wang Z., Lu L., Duan C., Wang X., Yang H. (2017). Morphological analysis of mitochondria for evaluating the toxicity of alpha-synuclein in transgenic mice and isolated preparations by atomic force microscopy. Biomed. Pharmacother..

[50] Malishev R., Kolusheva S., Jelinek R. (2019). Vesicle-Based Assays to Study Membrane Interactions of Amyloid Peptides. Methods. Mol. Biol..

[51] Stefanovic A.N., Stockl M.T., Claessens M.M., Subramaniam V. (2014). alpha-Synuclein oligomers distinctively permeabilize complex model membranes. FEBS J..

[52] Makin O.S., Atkins E., Sikorski P., Johansson J., Serpell L.C. (2005). Molecular basis for amyloid fibril formation and stability. Proc. Natl. Acad. Sci. USA.

[53] Freyssin A., Page G., Fauconneau B., Rioux Bilan A. (2018). Natural polyphenols effects on protein aggregates in Alzheimer’s and Parkinson’s prion-like diseases. Neural. Regen. Res..

[54] Uddin M.S., Kabir M.T., Niaz K., Jeandet P., Clement C., Mathew B., Rauf A., Rengasamy K.R.R., Sobarzo-Sanchez E., Ashraf G.M. (2020). Molecular Insight into the Therapeutic Promise of Flavonoids against Alzheimer’s Disease. Molecules.

[55] Zheng Q., Kebede M.T., Kemeh M.M., Islam S., Lee B., Bleck S.D., Wurfl L.A., Lazo N.D. (2019). Inhibition of the Self-Assembly of Abeta and of Tau by Polyphenols: Mechanistic Studies. Molecules.

[56] Caruana M., Hogen T., Levin J., Hillmer A., Giese A., Vassallo N. (2011). Inhibition and disaggregation of alpha-synuclein oligomers by natural polyphenolic compounds. FEBS Lett..

[57] Andrade S., Ramalho M.J., Loureiro J.A., Pereira M.C. (2019). Interaction of natural compounds with biomembrane models: A biophysical approach for the Alzheimer’s disease therapy. Colloid. Surf. B.

[58] Selvaraj S., Krishnaswamy S., Devashya V., Sethuraman S., Krishnan U.M. (2015). Influence of membrane lipid composition on flavonoid-membrane interactions: Implications on their biological activity. Prog. Lipid. Res..

[59] Grelle G., Otto A., Lorenz M., Frank R.F., Wanker E.E., Bieschke J. (2011). Black tea theaflavins inhibit formation of toxic amyloid-beta and alpha-synuclein fibrils. Biochemistry.

[60] Ren B., Liu Y., Zhang Y., Cai Y., Gong X., Chang Y., Xu L., Zheng J. (2018). Genistein: A Dual Inhibitor of Both Amyloid beta and Human Islet Amylin Peptides. ACS Chem. Neurosci..

[61] Bhasne K., Mukhopadhyay S. (2018). Formation of Heterotypic Amyloids: Alpha-Synuclein in Co-Aggregation. Proteomics.

[62] Young L.M., Mahood R.A., Saunders J.C., Tu L.H., Raleigh D.P., Radford S.E., Ashcroft A.E. (2015). Insights into the consequences of co-polymerisation in the early stages of IAPP and Abeta peptide assembly from mass spectrometry. Analyst.

[63] Pezzani R., Salehi B., Vitalini S., Iriti M., Zuniga F.A., Sharifi-Rad J., Martorell M., Martins N. (2019). Synergistic Effects of Plant Derivatives and Conventional Chemotherapeutic Agents: An Update on the Cancer Perspective. Medicina (Kaunas).

[64] Yuan H., Ma Q., Cui H., Liu G., Zhao X., Li W., Piao G. (2017). How Can Synergism of Traditional Medicines Benefit from Network Pharmacology?. Molecules.

[65] Evangleisti E., Cascella R., Becatti M., Marrazza G., Dobson C.M., Chiti F., Stefani M., Cecchi C. (2016). Binding affinity of amyloid oligomers to cellular membranes is a generic indicator of cellular dysfunction in protein misfolding diseases. Sci. Rep..

[66] Schmidt F., Levin J., Kamp F., Kretzschmar H., Giese A., Botzel K. (2012). Single-channel electrophysiology reveals a distinct and uniform pore complex formed by alpha-synuclein oligomers in lipid membranes. PLoS ONE.

[67] Bader B., Nubling G., Mehle A., Nobile S., Kretzschmar H., Giese A. (2011). Single particle analysis of tau oligomer formation induced by metal ions and organic solvents. Biochem. Biophys. Res. Commun..

[68] Kruger V., Deckers M., Hildenbeutel M., van der Laan M., Hellmers M., Dreker C., Preuss M., Herrmann J.M., Rehling P., Wagner R. (2012). The mitochondrial oxidase assembly protein1 (Oxa1) insertase forms a membrane pore in lipid bilayers. J. Biol. Chem..

[69] Horvath S.E., Daum G. (2013). Lipids of mitochondria. Prog. Lipid. Res..

[70] Gutsmann T., Heimburg T., Keyser U., Mahendran K.R., Winterhalter M. (2015). Protein reconstitution into freestanding planar lipid membranes for electrophysiological characterization. Nat. Protoc..

[71] Kayed R., Head E., Sarsoza F., Saing T., Cotman C.W., Necula M., Margol L., Wu J., Breydo L., Thompson J.L. (2007). Fibril specific, conformation dependent antibodies recognize a generic epitope common to amyloid fibrils and fibrillar oligomers that is absent in prefibrillar oligomers. Mol. Neurodegener..

