# Supplementary material for: Extract from the Marine Seaweed Padina pavonica Protects Mitochondrial Biomembranes from Damage by Amyloidogenic Peptides
Source: Molecules. 2021 Mar 7;26(5):1444. doi: 10.3390/molecules26051444 (PMC7962105; doi:10.3390/molecules26051444)
Supplement: Supplementary file 1 [file molecules-26-01444-s001.pdf]

Supplementary Table S1

The table shows the F-values and p-values pertaining to one-way ANOVA in Figures 1, 2, 3 and 5.

	**F (DFn, DFd)**	**P value**
**Figure 1A**	$F(4,12)=40.74$	< 0.0001
**Figure 1B**	$F(4,9)=12.91$	< 0.0009
**Figure 1C**	$F(2,12)=4.179$	0.0419
**Figure 2B**	$F(4,11)=13.19$	0.0004
**Figure 2C**	$F(2,8)=19.04$	0.0009
**Figure 2D**	$F(2,7)=22.96$	0.0008
**Figure 3A**	$F(2,29)=123.1$	<0.0001
**Figure 3B**	$F(2,4)=93.01$	0.0004
**Figure 5B**	$F(2,8)=86.68$	<0.0001
**Figure 5C**	$F(2,6)=180.7$	<0.0001
